# Supplementary material for: Role and mechanism of NCAPD3 in promoting malignant behaviors in gastric cancer
Source: Front Pharmacol. 2024 Apr 22;15:1341039. doi: 10.3389/fphar.2024.1341039 (PMC11070777; doi:10.3389/fphar.2024.1341039)
Supplement: Supplementary file 11 [file DataSheet2.ZIP › GSEA/Transcipt factor/my_analysis.Gsea.1599461814734/HOXA1_TARGET_GENES.html]

Details for gene set HOXA1\_TARGET\_GENES[GSEA]

|  || Dataset | filtered\_dataset.sample\_info.cls#WT\_versus\_NCAPD3\_MUT |
| Phenotype | sample\_info.cls#WT\_versus\_NCAPD3\_MUT |
| Upregulated in class | WT |
| GeneSet | HOXA1\_TARGET\_GENES |
| Enrichment Score (ES) | 0.23414408 |
| Normalized Enrichment Score (NES) | 1.7007611 |
| Nominal p-value | 0.01026393 |
| FDR q-value | 0.49424306 |
| FWER p-Value | 1.0 |
Table: GSEA Results Summary

  

Fig 1: Enrichment plot: HOXA1\_TARGET\_GENES      
 Profile of the Running ES Score & Positions of GeneSet Members on the Rank Ordered List

  

| SYMBOL | TITLE | RANK IN GENE LIST | RANK METRIC SCORE | RUNNING ES | CORE ENRICHMENT || 1 | 51312 | SLC25A37 | 12 | 1.077 | 0.0068 | Yes |
| 2 | 7571 | ZNF23 | 25 | 1.018 | 0.0128 | Yes |
| 3 | 119467 | CLRN3 | 42 | 0.944 | 0.0145 | Yes |
| 4 | 115209 | OMA1 | 45 | 0.932 | 0.0270 | Yes |
| 5 | 80746 | TSEN2 | 53 | 0.909 | 0.0351 | Yes |
| 6 | 91893 | FDXACB1 | 61 | 0.869 | 0.0427 | Yes |
| 7 | 117145 | THEM4 | 65 | 0.860 | 0.0533 | Yes |
| 8 | 153769 | SH3RF2 | 82 | 0.824 | 0.0532 | Yes |
| 9 | 9489 | PGS1 | 84 | 0.820 | 0.0648 | Yes |
| 10 | 112840 | WDR89 | 96 | 0.798 | 0.0682 | Yes |
| 11 | 55006 | TRMT61B | 101 | 0.782 | 0.0768 | Yes |
| 12 | 79109 | MAPKAP1 | 130 | 0.770 | 0.0666 | Yes |
| 13 | 26272 | FBXO4 | 133 | 0.767 | 0.0766 | Yes |
| 14 | 285343 | TCAIM | 134 | 0.765 | 0.0880 | Yes |
| 15 | 55900 | ZNF302 | 145 | 0.740 | 0.0914 | Yes |
| 16 | 8549 | LGR5 | 148 | 0.739 | 0.1009 | Yes |
| 17 | 51174 | TUBD1 | 153 | 0.730 | 0.1087 | Yes |
| 18 | 29940 | DSE | 174 | 0.703 | 0.1038 | Yes |
| 19 | 51018 | RRP15 | 192 | 0.685 | 0.1009 | Yes |
| 20 | 84190 | METTL25 | 195 | 0.684 | 0.1096 | Yes |
| 21 | 9456 | HOMER1 | 198 | 0.680 | 0.1182 | Yes |
| 22 | 4836 | NMT1 | 208 | 0.669 | 0.1212 | Yes |
| 23 | 55028 | C17orf80 | 222 | 0.659 | 0.1210 | Yes |
| 24 | 151636 | DTX3L | 233 | 0.649 | 0.1230 | Yes |
| 25 | 161779 | PGBD4 | 242 | 0.637 | 0.1264 | Yes |
| 26 | 8498 | RANBP3 | 251 | 0.629 | 0.1296 | Yes |
| 27 | 1161 | ERCC8 | 255 | 0.625 | 0.1366 | Yes |
| 28 | 55756 | INTS9 | 257 | 0.624 | 0.1452 | Yes |
| 29 | 83666 | PARP9 | 258 | 0.624 | 0.1546 | Yes |
| 30 | 54865 | GPATCH4 | 261 | 0.622 | 0.1623 | Yes |
| 31 | 25974 | MMACHC | 265 | 0.621 | 0.1693 | Yes |
| 32 | 25973 | PARS2 | 266 | 0.620 | 0.1786 | Yes |
| 33 | 23341 | DNAJC16 | 276 | 0.613 | 0.1808 | Yes |
| 34 | 54758 | KLHDC4 | 292 | 0.598 | 0.1782 | Yes |
| 35 | 84640 | USP38 | 303 | 0.589 | 0.1792 | Yes |
| 36 | 84946 | LTV1 | 319 | 0.583 | 0.1763 | Yes |
| 37 | 6715 | SRD5A1 | 325 | 0.578 | 0.1811 | Yes |
| 38 | 84135 | UTP15 | 330 | 0.576 | 0.1866 | Yes |
| 39 | 94107 | TMEM203 | 333 | 0.574 | 0.1937 | Yes |
| 40 | 2965 | GTF2H1 | 356 | 0.563 | 0.1851 | Yes |
| 41 | 10818 | FRS2 | 363 | 0.558 | 0.1888 | Yes |
| 42 | 57109 | REXO4 | 370 | 0.552 | 0.1924 | Yes |
| 43 | 2997 | GYS1 | 376 | 0.548 | 0.1968 | Yes |
| 44 | 1962 | EHHADH | 392 | 0.541 | 0.1932 | Yes |
| 45 | 54554 | WDR5B | 395 | 0.539 | 0.1998 | Yes |
| 46 | 10426 | TUBGCP3 | 402 | 0.535 | 0.2031 | Yes |
| 47 | 25917 | THUMPD3 | 412 | 0.530 | 0.2041 | Yes |
| 48 | 63929 | XPNPEP3 | 416 | 0.526 | 0.2096 | Yes |
| 49 | 55326 | AGPAT5 | 421 | 0.521 | 0.2143 | Yes |
| 50 | 54665 | RSBN1 | 429 | 0.516 | 0.2166 | Yes |
| 51 | 54931 | TRMT10C | 446 | 0.507 | 0.2118 | Yes |
| 52 | 148304 | C1orf74 | 447 | 0.506 | 0.2194 | Yes |
| 53 | 23192 | ATG4B | 452 | 0.505 | 0.2239 | Yes |
| 54 | 741 | ZNHIT2 | 453 | 0.503 | 0.2314 | Yes |
| 55 | 146691 | TOM1L2 | 472 | 0.490 | 0.2248 | Yes |
| 56 | 5245 | PHB | 481 | 0.486 | 0.2259 | Yes |
| 57 | 23116 | FAM179B | 482 | 0.486 | 0.2332 | Yes |
| 58 | 84261 | FBXW9 | 491 | 0.477 | 0.2341 | Yes |
| 59 | 54920 | DUS2 | 509 | 0.470 | 0.2280 | No |
| 60 | 90639 | COX19 | 535 | 0.454 | 0.2154 | No |
| 61 | 79080 | CCDC86 | 555 | 0.443 | 0.2074 | No |
| 62 | 5198 | PFAS | 570 | 0.437 | 0.2031 | No |
| 63 | 26164 | MTG2 | 585 | 0.433 | 0.1987 | No |
| 64 | 26057 | ANKRD17 | 589 | 0.431 | 0.2028 | No |
| 65 | 55181 | SMG8 | 602 | 0.422 | 0.1998 | No |
| 66 | 8021 | NUP214 | 611 | 0.417 | 0.1999 | No |
| 67 | 56339 | METTL3 | 613 | 0.417 | 0.2054 | No |
| 68 | 10061 | ABCF2 | 626 | 0.408 | 0.2022 | No |
| 69 | 54663 | WDR74 | 628 | 0.408 | 0.2075 | No |
| 70 | 10975 | UQCR11 | 632 | 0.406 | 0.2113 | No |
| 71 | 80110 | ZNF614 | 642 | 0.404 | 0.2103 | No |
| 72 | 26747 | NUFIP1 | 666 | 0.394 | 0.1984 | No |
| 73 | 54476 | RNF216 | 672 | 0.389 | 0.2004 | No |
| 74 | 23093 | TTLL5 | 684 | 0.386 | 0.1976 | No |
| 75 | 10607 | TBL3 | 698 | 0.380 | 0.1933 | No |
| 76 | 2932 | GSK3B | 714 | 0.367 | 0.1871 | No |
| 77 | 203068 | TUBB | 736 | 0.358 | 0.1762 | No |
| 78 | 8772 | FADD | 742 | 0.355 | 0.1777 | No |
| 79 | 58509 | CACTIN | 748 | 0.351 | 0.1790 | No |
| 80 | 23197 | FAF2 | 760 | 0.342 | 0.1757 | No |
| 81 | 23294 | ANKS1A | 768 | 0.339 | 0.1753 | No |
| 82 | 55178 | RNMTL1 | 771 | 0.337 | 0.1788 | No |
| 83 | 4869 | NPM1 | 772 | 0.336 | 0.1838 | No |
| 84 | 9238 | TBRG4 | 786 | 0.330 | 0.1787 | No |
| 85 | 158 | ADSL | 796 | 0.326 | 0.1766 | No |
| 86 | 39 | ACAT2 | 799 | 0.322 | 0.1799 | No |
| 87 | 79590 | MRPL24 | 800 | 0.320 | 0.1847 | No |
| 88 | 3704 | ITPA | 809 | 0.314 | 0.1832 | No |
| 89 | 51575 | ESF1 | 812 | 0.309 | 0.1863 | No |
| 90 | 8904 | CPNE1 | 814 | 0.308 | 0.1901 | No |
| 91 | 51373 | MRPS17 | 817 | 0.306 | 0.1931 | No |
| 92 | 10528 | NOP56 | 837 | 0.285 | 0.1827 | No |
| 93 | 10856 | RUVBL2 | 847 | 0.257 | 0.1796 | No |
| 94 | 4071 | TM4SF1 | 851 | -0.251 | 0.1810 | No |
| 95 | 667 | DST | 886 | -0.303 | 0.1592 | No |
| 96 | 2150 | F2RL1 | 887 | -0.304 | 0.1637 | No |
| 97 | 3164 | NR4A1 | 912 | -0.341 | 0.1502 | No |
| 98 | 3475 | IFRD1 | 935 | -0.360 | 0.1386 | No |
| 99 | 10938 | EHD1 | 1019 | -0.419 | 0.0805 | No |
| 100 | 6590 | SLPI | 1062 | -0.453 | 0.0547 | No |
| 101 | 25976 | TIPARP | 1063 | -0.454 | 0.0615 | No |
| 102 | 27111 | SDCBP2 | 1070 | -0.457 | 0.0637 | No |
| 103 | 100534599 | ISY1-RAB43 | 1082 | -0.462 | 0.0621 | No |
| 104 | 1604 | CD55 | 1090 | -0.468 | 0.0637 | No |
| 105 | 7378 | UPP1 | 1111 | -0.484 | 0.0555 | No |
| 106 | 51105 | PHF20L1 | 1140 | -0.506 | 0.0413 | No |
| 107 | 7444 | VRK2 | 1145 | -0.509 | 0.0459 | No |
| 108 | 1032 | CDKN2D | 1156 | -0.519 | 0.0459 | No |
| 109 | 54407 | SLC38A2 | 1182 | -0.553 | 0.0348 | No |
| 110 | 6430 | SRSF5 | 1183 | -0.553 | 0.0431 | No |
| 111 | 9113 | LATS1 | 1201 | -0.577 | 0.0386 | No |
| 112 | 57649 | PHF12 | 1212 | -0.588 | 0.0396 | No |
| 113 | 4651 | MYO10 | 1213 | -0.588 | 0.0484 | No |
| 114 | 10221 | TRIB1 | 1229 | -0.604 | 0.0459 | No |
| 115 | 22822 | PHLDA1 | 1255 | -0.643 | 0.0361 | No |
| 116 | 83931 | STK40 | 1281 | -0.669 | 0.0268 | No |
| 117 | 57553 | MICAL3 | 1318 | -0.732 | 0.0098 | No |
| 118 | 4233 | MET | 1358 | -0.821 | -0.0081 | No |
| 119 | 115123 | MARCH3 | 1360 | -0.828 | 0.0035 | No |
| 120 | 6782 | HSPA13 | 1394 | -1.064 | -0.0061 | No |
| 121 | 3329 | HSPD1 | 1402 | -1.183 | 0.0062 | No |
Table: GSEA details [plain text format]

  

Fig 2: HOXA1\_TARGET\_GENES      
 Blue-Pink O' Gram in the Space of the Analyzed GeneSet

  

Fig 3: HOXA1\_TARGET\_GENES: Random ES distribution      
 Gene set null distribution of ES for **HOXA1\_TARGET\_GENES**
